# Omission of Axillary Lymph Node Dissection in Breast Cancer Patients with 1–2 Positive Sentinel Lymph Nodes: A Multicenter Real-World Cohort Study in a Chinese Population

**DOI:** 10.3390/curroncol33050247

**Published:** 2026-04-27

**Authors:** Chengye Hong, Jianhui Chen, Yongwu Chen, Liangqiang Li, Xianqiang Du, Debo Chen, Weibin Lian

**Affiliations:** 1 Department of Breast Surgery, Quanzhou First Hospital Affiliated to Fujian Medical University, Quanzhou 362000, China; hongcy01@fjmu.edu.cn (C.H.); lilq880620@163.com (L.L.); dxq20140528@hotmail.com (X.D.); 2 Department of Breast Surgery, Zhangzhou Affiliated Hospital of Fujian Medical University, Zhangzhou 363000, China; drchen008@fjmu.edu.cn; 3 Department of Breast Surgery, Longyan Second Hospital, Longyan 364000, China; yongwuchen520@163.com

**Keywords:** breast cancer, sentinel lymph node biopsy, axillary lymph node dissection, Z0011 criteria, real-world study

## Abstract

The optimal management of patients with limited sentinel lymph node metastasis in breast cancer, particularly regarding whether to perform additional axillary surgery, continues to be an area of clinical uncertainty in routine practice. This multicenter retrospective cohort study aimed to evaluate adherence to ACOSOG Z0011 criteria and the oncological safety of omitting ALND in a Chinese population. We included 462 women with clinical stage T1–2N0 breast cancer who underwent breast-conserving surgery and were found to have 1–2 positive SLNs between January 2013 and December 2021. All patients received adjuvant radiotherapy and systemic therapy. Patients underwent either sentinel lymph node biopsy alone (SLNB; n = 274, 59.3%) or SLNB followed by ALND (n = 188, 40.7%). Propensity score matching (1:1) was applied to balance baseline characteristics, yielding 152 matched pairs. Disease-free survival (DFS) was the primary endpoint. No significant difference in DFS was observed between the SLNB alone and SLNB + ALND groups in either the overall cohort or the matched cohort. Multivariable Cox regression analysis confirmed that the type of axillary surgery was not independently associated with DFS in patients with 1–2 positive SLNs treated with breast-conserving surgery. Logistic regression analysis indicated that surgeons were more likely to perform ALND in patients with a higher SLN tumor burden; compared with micrometastasis, macrometastasis in 1–2 SLNs and a sentinel lymph node metastasis ratio greater than one-third were significantly associated with the selection of ALND. These findings suggest that omission of ALND was not associated with a statistically significant difference in DFS and provide real-world evidence supporting the applicability of Z0011-based axillary management in the Chinese population; however, given the observational design and potential for residual confounding, these results should be interpreted with caution.

## Simple Summary

In early-stage breast cancer, removing lymph nodes from the armpit has long been a standard procedure but may cause complications such as arm swelling and reduced mobility. Recent studies suggest that some patients with limited lymph node involvement may safely avoid this more extensive surgery. In this multicenter study of Chinese patients, we compared outcomes between those who underwent sentinel lymph node biopsy alone and those who received additional lymph node removal. We found no difference in disease-free survival between the two groups, suggesting that less invasive surgery may be a safe option for selected patients. These findings support a shift toward reducing surgical burden and may help guide future clinical practice and decision-making.

## 1. Introduction

The management of the axilla in early-stage breast cancer has undergone a paradigm shift over the past two decades, particularly in patients with clinically node-negative (cN0) disease and limited sentinel lymph node (SLN) metastases. This evolution reflects a broader movement toward treatment de-escalation, aiming to reduce surgical morbidity without compromising oncologic safety. Historically, axillary lymph node dissection (ALND) was routinely performed for both staging and locoregional control. However, accumulating evidence from randomized clinical trials has challenged this paradigm and supported less invasive approaches in carefully selected patient populations. The International Breast Cancer Study Group (IBCSG) 23-01 trial demonstrated that, in patients with SLN micrometastases (≤2 mm) and tumor size ≤ 5 cm, omission of ALND was non-inferior to ALND in terms of disease-free survival (DFS) and overall survival [1,2]. Subsequently, the ACOSOG Z0011 trial significantly reshaped axillary management in patients with T1–T2 invasive breast cancer, clinically node-negative disease, and 1–2 positive SLNs undergoing breast-conserving surgery with whole-breast irradiation. The trial showed no significant differences in locoregional recurrence or survival outcomes between SLNB alone and completion ALND groups [3]. These findings were incorporated into major clinical guidelines. The American Society of Clinical Oncology (ASCO) guideline update in 2017 recommends against routine completion ALND in patients with T1–T2 cN0 breast cancer with 1–2 positive SLNs who meet Z0011 eligibility criteria, particularly in the context of breast-conserving therapy and adjuvant systemic treatment [4]. Similar recommendations are endorsed by the National Comprehensive Cancer Network (NCCN), which supports omission of ALND in Z0011-eligible patients receiving breast-conserving surgery and whole-breast irradiation. Despite high-level evidence and guideline concordance, real-world implementation remains suboptimal. Data from the National Cancer Database (NCDB) indicate that up to approximately 50% of patients meeting Z0011 criteria still undergo ALND, reflecting persistent variability in clinical practice and potential overtreatment [5]. This gap between evidence and practice has also been observed in non-Western populations. A single-center prospective study in China reported that 81% (115/142) of patients meeting Z0011 criteria underwent SLNB alone; however, oncologic outcomes could not be robustly assessed due to limited follow-up duration [6]. These findings highlight both improving adoption and ongoing uncertainty regarding long-term outcomes in real-world Asian cohorts.

In this context, we conducted a large multicenter retrospective cohort study in China to evaluate adherence to Z0011 criteria and to assess the oncologic safety of sentinel lymph node biopsy (SLNB) alone compared with completion ALND in patients with 1–2 positive SLNs. Additionally, we aimed to identify clinicopathological and treatment-related factors associated with the selection of axillary surgical strategies, thereby providing real-world evidence to inform de-escalation strategies in routine clinical practice.

## 2. Methods

### 2.1. Patients and Data Source

This retrospective multicenter cohort study included women diagnosed with early-stage invasive breast cancer (clinical T1–2N0) who underwent breast-conserving surgery between 1 January 2013 and 31 December 2021. The study population was derived from the breast centers of three comprehensive hospitals in China: Shanghai Jiao Tong University School of Medicine Affiliated Ruijin Hospital (n = 259), Quanzhou First Hospital Affiliated to Fujian Medical University (n = 134), and Zhangzhou Hospital Affiliated to Fujian Medical University (n = 69). Eligible patients fulfilled the ACOSOG Z0011 criteria: (1) clinically node-negative disease (cN0), (2) 1–2 metastatic SLNs identified after surgery, and (3) receipt of whole-breast radiotherapy and appropriate systemic therapy. Patients who received neoadjuvant therapy, had male breast cancer, had prior invasive malignancies, or had incomplete pathological, treatment, or follow-up data were excluded. Study inclusion and analytic classification were based on final permanent paraffin-embedded pathology results rather than intraoperative frozen section findings, in accordance with standard pathological practice. The study was approved by the independent ethics committees of Quanzhou First Hospital and conducted in accordance with the Declaration of Helsinki. The requirement for informed consent was waived due to the retrospective nature of the study.

### 2.2. Axillary Surgical Management and Pathological Assessment

SLNB was performed using blue dye mapping according to institutional standards. Resected sentinel lymph nodes were routinely evaluated by intraoperative frozen section. When metastatic involvement was identified, completion ALND was performed according to institutional practice. However, final study group allocation (SLNB alone versus SLNB + ALND) and all statistical analyses were determined according to permanent paraffin-embedded pathology findings rather than frozen section results. In cases of discordance between intraoperative frozen section and final paraffin pathology findings, the permanent pathology result was considered definitive for nodal classification in the study. Following receipt of the final pathology report, the decision to proceed with completion ALND was primarily influenced by objective indicators of nodal burden, including the identification of macrometastases on frozen sections, suspected extranodal extension, or a high ratio of positive-to-removed sentinel nodes identified intraoperatively. To mitigate the inherent selection bias associated with these surgical decisions, we utilized propensity score matching (PSM) and multivariable regression models to balance nodal tumor burden and other clinicopathological factors across comparison groups. Micrometastasis was defined as tumor deposits greater than 0.2 mm and not exceeding 2 mm in greatest dimension, whereas macrometastasis was defined as tumor deposits larger than 2 mm, consistent with standard pathological criteria.

### 2.3. Definition of Nodal Yield and Axillary Tumor Burden

Sentinel lymph node tumor burden at the time of SLNB was characterized using: Number of sentinel lymph node metastases (NSLNM) and proportion of positive sentinel lymph nodes (PSLNM), calculated as the ratio of positive SLNs to total SLNs removed. These variables reflect intraoperative nodal involvement prior to any completion ALND and were used to evaluate surgical decision-making patterns. For patients who underwent completion ALND, total axillary tumor burden was defined as the total number of metastatic axillary lymph nodes identified on final pathology, including both sentinel and non-sentinel lymph nodes. The category “≥3 positive lymph nodes” refers specifically to the total number of metastatic axillary lymph nodes identified after completion ALND, rather than sentinel lymph nodes alone. In the SLNB + ALND group, the median number of axillary lymph nodes removed was 22 (range 10–35), and the median number of positive nodes was 4 (range 1–15). Patients were further categorized into 3 nodes (n = 136), 4–9 nodes (n = 39), and ≥10 nodes (n = 13).

### 2.4. Adjuvant Radiotherapy

All patients received adjuvant whole-breast irradiation after breast-conserving surgery. Radiotherapy techniques were based on institutional practice patterns during the study period. Tangential field irradiation was routinely applied, which may provide incidental coverage of level I–II axillary lymph nodes. Detailed information regarding elective regional nodal irradiation (including supraclavicular or axillary level III fields) was not consistently available in the dataset.

### 2.5. Outcomes and Follow-Up

The primary endpoint of this study was DFS, defined as the time from the date of breast-conserving surgery to the first documented event of locoregional recurrence, distant metastasis, or death from any cause. Follow-up duration was calculated from the date of surgery to the date of last contact or event occurrence.

### 2.6. Statistical Analysis

All statistical analyses were performed using R software (version 4.2.3). A two-sided *p* value < 0.05 was considered statistically significant. Baseline characteristics between the SLNB and ALND groups were compared using chi-square or Fisher’s exact tests for categorical variables. Survival outcomes were estimated using the Kaplan–Meier curve and compared with the log-rank test. To minimize confounding due to non-random treatment allocation, propensity score matching (PSM) was conducted using a 1:1 nearest-neighbor matching algorithm with a caliper width of 0.2 standard deviations of the logit of the propensity score. PSM was conducted using the “MatchIt” package, and survival analyses were performed using the “survival” and “survminer” packages. A fixed random seed (set.seed(100)) was applied to ensure reproducibility of the matching process. Covariates included in the propensity model were molecular subtype, PSLNM, NSLNM and chemotherapy. Covariate balance after matching was assessed using standardized mean differences (SMD), with values <0.1 considered indicative of adequate balance. The baseline characteristics before and after matching are presented in Supplementary Table S1. After matching, the demographic and pathological characteristics, as well as the treatment baseline of the two groups, achieved balance (The baseline characteristics after matching are presented in Supplementary Table S1). To identify factors influencing the choice of axillary surgical management (SLNB + ALND vs. SLNB alone), multivariable logistic regression analysis was performed. Variables included in the multivariable Cox regression models were selected based on clinical relevance and prior literature, including age, clinical T stage, molecular subtype, Ki-67 index, lymphovascular invasion (LVI), and nodal burden indicators. This approach was used to ensure appropriate adjustment for potential confounders while avoiding overfitting. Missing data were handled using a complete-case analysis approach. In logistic regression models, micrometastasis was selected as the reference category for NPSLN, as it represents the lowest nodal burden subgroup. Consequently, odds ratios for patients with one or two macrometastatic nodes appear numerically small, reflecting increased likelihood of undergoing ALND relative to the micrometastasis group. All regression analyses were conducted based on final paraffin pathology data to ensure consistent biological classification across comparison groups.

## 3. Results

### 3.1. Patients

In this study, we evaluated the baseline clinicopathological characteristics of 462 Chinese breast cancer patients, stratified into two surgical groups: SLNB alone (n = 274) and SLNB followed by ALND (n = 188). As summarized in Table 1, the proportion of lymph node micrometastasis was significantly higher in the SLNB alone group than in the SLNB + ALND group (18.25% vs. 1.60%). Conversely, patients with two metastatic sentinel lymph nodes were more likely to undergo SLNB + ALND (24.47% vs. 10.95%). Moreover, the proportion of sentinel lymph node metastasis (PSLNM) ≤ 1/3 was significantly greater in the SLNB alone group compared with the SLNB + ALND group (79.93% vs. 54.26%, *p* < 0.001). A significant difference was also observed in chemotherapy administration: 7.45% of patients in the SLNB + ALND group received chemotherapy, compared with 17.15% in the SLNB alone group (*p* = 0.002). In contrast, the distribution of Ki67 proliferation index and molecular subtypes was comparable between the two groups. No statistically significant differences were observed in lymphovascular invasion (LVI) or clinical T stage.

### 3.2. Survival Analysis

**Figure 1:**
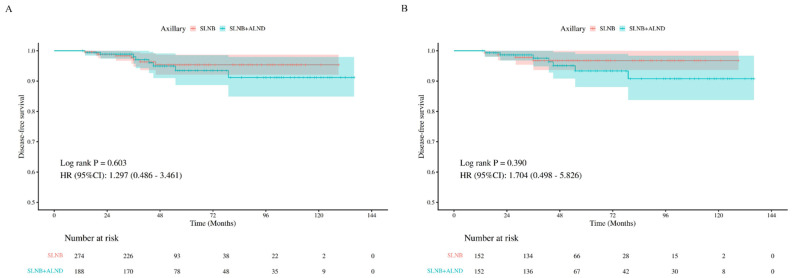
**Disease-free survival (DFS) according to axillary surgical strategy in patients with 1–2 positive sentinel lymph nodes undergoing breast-conserving surgery.** Kaplan–Meier curves compare DFS between patients treated with sentinel lymph node biopsy (SLNB) alone and those who underwent SLNB plus axillary lymph node dissection (ALND), shown for the overall cohort before propensity score matching (PSM) (**A**) and for the matched cohort after 1:1 PSM (**B**); matching variables included molecular subtype, proportion of sentinel lymph node metastasis (PSLNM), the number of sentinel lymph node metastases (NSLNM) and chemotherapy. Note: Kaplan-Meier curves represent unadjusted survival data; please refer to Table 2 for the multivariable-adjusted hazard ratios.

During a median follow-up of 49 months, a total of 16 DFS events were observed in the entire cohort, with an equal distribution between the SLNB group (n = 8) and the SLNB + ALND group (n = 8). In the SLNB group, events included 3 locoregional recurrences and 5 distant metastases, with no deaths reported. In the SLNB + ALND group, there was 1 locoregional recurrence, 6 distant metastases, and 1 death. Kaplan–Meier survival analysis was first performed to compare DFS between the SLNB and SLNB + ALND groups (Figure 1A). No statistically significant difference was observed in this unadjusted analysis (*p* = 0.603). Subsequently, PSM was conducted to generate well-balanced cohorts of patients who underwent SLNB alone (n = 152) and SLNB + ALND (n = 152). Consistent with the findings in the overall cohort, no significant difference in DFS was observed between the matched groups (*p* = 0.390, Figure 1B). Finally, univariable and multivariable Cox proportional hazards regression analyses were performed. The results likewise indicated that the type of axillary management was not significantly associated with DFS, with a multivariable-adjusted HR of 1.32 (95% CI: 0.42–4.12, *p* = 0.635; Table 2). No significant violation of the proportional hazards assumption was observed for any variable included in the Cox regression models.

### 3.3. Factors Influencing Decision-Making

We further investigated factors associated with the choice of axillary surgical management (Table 3). The NSLNM was a significant determinant of surgical strategy. Compared with the micrometastasis reference group, patients with one macrometastatic SLN had an odds ratio (OR) of 0.08 (95% CI: 0.03–0.27, *p* < 0.001), and those with two macrometastatic SLNs had an OR of 0.04 (95% CI: 0.01–0.14, *p* < 0.001). These markedly low OR values indicate a substantially higher likelihood of undergoing ALND rather than SLNB alone among patients with one or two macrometastatic nodes. In addition, the PSLNM was significantly associated with axillary management. Patients with PSLNM > 1/3 had an OR of 0.30 (95% CI: 0.20–0.45, *p* < 0.001) in univariable analysis, and this association remained robust in multivariable analysis (OR = 0.32, 95% CI: 0.19–0.54, *p* < 0.001).

## 4. Discussion

This multicenter real-world study evaluated the oncologic safety of omitting completion ALND in Chinese patients who met the ACOSOG Z0011 criteria. Our findings demonstrated that SLNB alone was not associated with inferior DFS compared with SLNB + ALND, both in the overall cohort and after rigorous propensity score matching and multivariable adjustment. These results provide real-world evidence supporting the feasibility and oncologic safety of axillary surgical de-escalation in Chinese patients. Importantly, these findings should be interpreted within the therapeutic paradigm established by the Z0011 trial. The safety of omitting ALND was not predicated solely on limited nodal burden but rather on the comprehensive integration of breast-conserving surgery, whole-breast irradiation, and systemic therapy. In our cohort, all patients received adjuvant whole-breast radiotherapy following breast-conserving surgery. Standard tangential field irradiation, routinely applied in clinical practice, may provide incidental coverage of level I–II axillary lymph nodes, thereby contributing to regional disease control. Although detailed radiotherapy contouring data were not uniformly available, the low rates of locoregional and axillary recurrence observed in our study suggest that adequate regional control was maintained.

SLNB has become the standard approach for axillary staging because it significantly reduces surgical morbidity compared with ALND. The paradigm shift toward de-escalation was primarily driven by the Z0011 trial, which demonstrated non-inferior survival outcomes in patients undergoing SLNB alone. Similarly, the IBCSG 23-01 trial showed that omission of ALND in patients with sentinel lymph node micrometastasis did not compromise DFS [2]. Furthermore, the landmark AMAROS trial demonstrated that axillary radiotherapy provides regional control comparable to ALND for patients with positive SLNs in T1–2 tumors, but with significantly lower morbidity. This supports the premise that surgical de-escalation is oncologically safe when integrated with appropriate regional radiotherapy, as applied in our cohort [7]. More recently, the SENOMAC trial further confirmed the safety of avoiding ALND in patients with one or two macrometastatic SLNs, reporting comparable 5-year recurrence-free survival [8]. Importantly, patient-reported outcomes from the SENOMAC trial demonstrated significantly better Lymph-ICF domain scores in physical function, mental function, and mobility activities among patients undergoing SLNB alone, highlighting the functional advantages of minimizing axillary surgery [9]. Additionally, a systematic review by Lai et al. highlights that omitting ALND in older breast cancer patients with limited SLN involvement does not adversely affect overall survival [10]. These findings are corroborated by the ASCO guidelines, which recommend against routine ALND for patients with 1–2 positive SLNs who will receive breast-conserving surgery and radiation therapy [11]. The rationale is that the survival outcomes for these patients are comparable to those who undergo ALND, thus supporting a less invasive approach.

However, it is essential to recognize that individual patient characteristics can significantly influence clinical outcomes. Wang et al. highlighted that while omitting ALND is generally recommended, it may not be safe for all patients. Their research identified risk factors associated with non-SLN metastases, suggesting that a predictive model could help identify patients who might still benefit from ALND. This model included factors such as tumor size and the number of positive SLNs, which can guide clinical decision-making [12]. Moreover, a study by Yu et al. developed nomograms to predict non-SLN metastases in patients with 1–2 positive SLNs, demonstrating that a significant proportion of these patients could safely avoid ALND [13]. The findings suggest that careful patient selection based on specific pathological features can lead to better outcomes and reduced morbidity.

Nevertheless, individual tumor burden remains a critical determinant in surgical decision-making. Our analysis demonstrated that both the number of positive SLNs (NPSLN) and the proportion of sentinel lymph node metastasis (PSLNM) independently influenced the likelihood of undergoing ALND. Patients with one or two macrometastatic SLNs and those with PSLNM > 1/3 were significantly more likely to receive ALND, reflecting a tendency among surgeons to escalate axillary treatment when nodal tumor burden appears higher. These findings are consistent with previous predictive modeling studies suggesting that tumor size, nodal burden, and related pathological features are associated with non-sentinel lymph node involvement and may influence the perceived need for completion ALND. Notably, this study is among the few to systematically incorporate PSLNM as a quantitative indicator in axillary decision-making. By introducing this proportion-based metric, which may more accurately reflect relative nodal tumor burden than absolute node counts alone, our analysis provides additional insight into how surgeons interpret pathological information in real-world practice. This quantitative perspective may facilitate more individualized and risk-adapted axillary management strategies in the future.

The strengths of this study include its multicenter design involving three breast cancer centers, a relatively large sample size, and the application of multiple complementary statistical approaches, including PSM and multivariable regression analyses, which enhance the external validity and generalizability of our findings within the context of real-world clinical practice in China. Several limitations should also be acknowledged. First, given the retrospective and observational nature of the study, residual confounding cannot be completely excluded. Although PSM was performed to reduce selection bias, the propensity model included a limited number of variables. Attempts to incorporate a broader set of clinically relevant covariates resulted in poor overlap between treatment groups and unstable matching, reflecting inherent differences in baseline risk profiles. Therefore, a parsimonious propensity score model was adopted to achieve stable matching while preserving sample size. Importantly, axillary surgical decision-making in real-world practice is influenced by multiple prognostic factors, leading to inherent selection bias that cannot be fully eliminated through matching alone. To further mitigate this limitation, multivariable Cox regression analyses adjusting for key clinical covariates were performed, and the results remained consistent across different analytical approaches, including the overall cohort and the matched cohort. These findings suggest that omission of ALND was not associated with inferior disease-free survival in this population when Z0011 criteria were appropriately applied. In addition, variability in surgical expertise, radiotherapy techniques, and institutional treatment protocols across centers may have introduced heterogeneity. Furthermore, the median follow-up duration of 49 months, while clinically meaningful, may be insufficient to fully capture long-term recurrence patterns, particularly for late events. Therefore, the findings of this study should be interpreted with caution and warrant validation in prospective studies, particularly in Asian populations.

## Figures and Tables

**Table 1:** Baseline Characteristics.

Variables	Total (n = 462)	SLNB + ALND (n = 188)	SLNB (n = 274)	*p*
**Age, n (%)**				0.750
≤50 y	249 (53.90)	103 (54.79)	146 (53.28)	
>50 y	213 (46.10)	85 (45.21)	128 (46.72)	
**BMI, n (%)**				0.259
<25 kg/m^2^	335 (72.51)	131 (69.68)	204 (74.45)	
≥25 kg/m^2^	127 (27.49)	57 (30.32)	70 (25.55)	
**Grade, n (%)**				0.196
I	26 (5.63)	15 (7.98)	11 (4.01)	
II	301 (65.15)	114 (60.64)	187 (68.25)	
III	106 (22.94)	47 (25.00)	59 (21.53)	
Unknow	29 (6.28)	12 (6.38)	17 (6.20)	
**LVI, n (%)**				0.278
Absent	398 (86.15)	158 (84.04)	240 (87.59)	
Present	64 (13.85)	30 (15.96)	34 (12.41)	
**Chemotherapy, n (%)**				0.002
No	61 (13.20)	14 (7.45)	47 (17.15)	
Yes	401 (86.80)	174 (92.55)	227 (82.85)	
**NSLNM, n (%)**				<0.001
Micrometastases	53 (11.47)	3 (1.60)	50 (18.25)	
1	333 (72.08)	139 (73.94)	194 (70.80)	
2	76 (16.45)	46 (24.47)	30 (10.95)	
**PSLNM, n (%)**				<0.001
≤1/3	321 (69.48)	102 (54.26)	219 (79.93)	
>1/3	141 (30.52)	86 (45.74)	55 (20.07)	
**Ki67, n (%)**				0.855
≤14%	162 (35.06)	65 (34.57)	97 (35.40)	
>14%	300 (64.94)	123 (65.43)	177 (64.60)	
**Molecular Subtype, n (%)**				0.831
HR+/HER2−	403 (87.23)	163 (86.70)	240 (87.59)	
HER2+	49 (10.61)	20 (10.64)	29 (10.58)	
TNBC	10 (2.16)	5 (2.66)	5 (1.82)	
**Clinical T stage, n (%)**				0.333
T1	326 (70.56)	128 (68.09)	198 (72.26)	
T2	136 (29.44)	60 (31.91)	76 (27.74)	
**Anti-HER2 therapy, n (%)**				0.614
No	412 (89.18)	166 (88.30)	246 (89.78)	
Yes	50 (10.82)	22 (11.70)	28 (10.22)	
**Endocrine therapy, n (%)**				0.071
No	16 (3.46)	10 (5.32)	6 (2.19)	
Yes	446 (96.54)	178 (94.68)	268 (97.81)	

**Abbreviations:** SLNB = sentinel lymph node biopsy, ALND = axillary lymph node dissection, BMI = body mass index, LVI = lymphovascular invasion, NSLNM = number of sentinel lymph node metastases, PSLNM = proportion of sentinel lymph node metastasis, HR+/HER2− = hormone receptor-positive and human epidermal growth factor receptor 2–negative, TNBC = triple-negative breast cancer.

**Table 2:** Univariate and multivariate Cox regression analyses of factors associated with disease-free survival in patients with 1–2 positive SLNs undergoing breast-conserving surgery.

Variables	Univariate			Multivariable		
	HR	95% CI	*p* Value	HR	95% CI	*p* Value
**Axillary treatment**						
SLNB	Ref			Ref		
SLNB + ALND	1.3	0.49~3.46	0.604	1.32	0.42~4.12	0.635
**BMI**						
<25 kg/m^2^	Ref			Ref		
≥25 kg/m^2^	0.65	0.18~2.28	0.499	0.6	0.16~2.18	0.433
**Age**						
≤50 y	Ref			Ref		
>50 y	0.49	0.17~1.42	0.133	0.56	0.19~1.72	0.313
**Grade**						
I	Ref			Ref		
II	0.43	0.05~3.72	0.446	0.38	0.04~3.50	0.391
III	1. 75	0.22~12.88	0.595	1.73	0.18~17.13	0.637
**LVI**						
Absent	Ref			Ref		
Present	3.12	1.08~9.06	0.036	3.19	1.01~10.04	0.047
**No. of positive lymph node**						
1	Ref			Ref		
2	0.61	0.14~2.72	0.516	0.45	0.09~2.18	0.321
≥3	2.66	0.59~11.9	0.201	2.59	0.43~15.58	0.297
**Clinical T stage**						
T1	Ref			Ref		
T2	0.54	0.15~1.91	0.341	0.31	0.08~1.15	0.081
**Subtype**						
HR+/HER2−	Ref			Ref		
Her2+	2.05	0.58~7.27	0.267	1.24	0.32~4.77	0.757
TNBC	2.29	0.29~17.94	0.429	1.56	0.18~13.86	0.689
**Ki67**						
≤14%	Ref			Ref		
>14%	2.55	0.73~8.95	0.144	1.71	0.43~6.82	0.445

**Abbreviations:** HR = hazard ratio, CI = confidence interval, SLNB = sentinel lymph node biopsy, ALND = axillary lymph node dissection, BMI = body mass index, LVI = lymphovascular invasion, HR+/HER2− = hormone receptor-positive and human epidermal growth factor receptor 2–negative, TNBC = triple-negative breast cancer. **Note:** ≥3 positive lymph nodes indicates total axillary metastatic nodes identified after completion ALND.

**Table 3:** Univariate and multivariate analysis of impact factors for the surgical management of the axilla in patients with 1–2 positive SLNs undergoing breast-conserving surgery.

Variables	Univariate		Multivariate	
	OR (95% CI)	*p*	OR (95% CI)	*p*
**Age**				
≤50 y	1.00 (Reference)			
>50 y	1.06 (0.73~1.54)	0.750		
**BMI**				
<25 kg/m^2^	1.00 (Reference)			
≥25 kg/m^2^	0.79 (0.52~1.19)	0.260		
**Clinical T stage**				
T1	1.00 (Reference)			
T2	0.82 (0.55~1.23)	0.333		
**NSLNM**				
micrometastases	1.00 (Reference)			
1	0.08 (0.03~0.27)	<0.001	0.08 (0.03~0.28)	<0.001
2	0.04 (0.01~0.14)	<0.001	0.08 (0.02~0.31)	<0.001
**PSLNM**				
≤1/3	1.00 (Reference)		1.00 (Reference)	
>1/3	0.30 (0.20~0.45)	<0.001	0.32 (0.19~0.54)	<0.001
**Ki67**				
<14%	1.00 (Reference)			
≥14%	0.96 (0.65~1.42)	0.855		
**Subtype**				
HR+/HER2−	1.00 (Reference)			
HER2+	0.98 (0.54~1.80)	0.960		
TNBC	0.68 (0.19~2.38)	0.546		

Abbreviations: SLNs = sentinel lymph nodes, OR = odds ratio, CI = confidence interval, BMI = body mass index, NSLNM = number of sentinel lymph node metastases, PSLNM = proportion of sentinel lymph node metastasis, HR+/HER2− = hormone receptor-positive and human epidermal growth factor receptor 2–negative, TNBC = triple-negative breast cancer.

## Data Availability

The data supporting all tables and figures in this published article are not publicly available to protect patient privacy but can be accessed from the corresponding author on request.

## Supplementary Materials

The following supporting information can be downloaded at https://www.mdpi.com/article/10.3390/curroncol33050247/s1. Table S1. Baseline characteristics before and after propensity score matching.
