# Supplementary material for: Omission of Axillary Lymph Node Dissection in Breast Cancer Patients with 1–2 Positive Sentinel Lymph Nodes: A Multicenter Real-World Cohort Study in a Chinese Population
Source: Curr Oncol. 2026 Apr 27;33(5):247. doi: 10.3390/curroncol33050247 (PMC13204892; doi:10.3390/curroncol33050247)
Supplement: Supplementary file 1 [file curroncol-33-00247-s001.zip › curroncol-4223842-supplementary.pdf]

Supplementary Table S1. Baseline Characteristics Before and After Propensity Score Matching.

Variable	Before PSM			After PSM		
	SLNB (n = 274)	SLNB+ALND (n = 188)	P	SLNB (n = 152)	SLNB+ALND (n = 152)	P
**Subtype, n (%)**			0.831			1.000
HER2+	29 (10.58)	20 (10.64)		20 (13.16)	20 (13.16)	
HR+/HER2-	240 (87.59)	163 (86.70)		129 (84.87)	129 (84.87)	
TNBC	5 (1.82)	5 (2.66)		3 (1.97)	3 (1.97)	
**Chemotherapy, n (%)**			0.002			1.000
No	47 (17.15)	14 (7.45)		13 (8.55)	13 (8.55)	
Yes	227 (82.85)	174 (92.55)		139 (91.45)	139 (91.45)	
**NSLNM, n (%)**			<0.001			0.062
Micrometastases	50 (18.25)	3 (1.60)		3 (1.97)	3 (1.97)	
1	194 (70.80)	139 (73.94)		126 (82.89)	139 (91.45)	
2	30 (10.95)	46 (24.47)		23 (15.13)	10 (6.58)	
**PSLNM, n (%)**			<0.001			1.000
≤1/3	219 (79.93)	102 (54.26)		102 (67.11)	102 (67.11)	
>1/3	55 (20.07)	86 (45.74)		50 (32.89)	50 (32.89)	
